# A Method of Sky Ripple Residual Nonuniformity Reduction for a Cooled Infrared Imager and Hardware Implementation

**DOI:** 10.3390/s17051070

**Published:** 2017-05-08

**Authors:** Yiyang Li, Weiqi Jin, Shuo Li, Xu Zhang, Jin Zhu

**Affiliations:** School of Optoelectronics, Beijing Institute of Technology, Key Laboratory of Photo-electronic Imaging Technology and System, Ministry of Education of China, Beijing 100081, China; neighborhoodlyy@163.com (Y.L.); lishuo93@163.com (S.L.); zhangxu610521@163.com (X.Z.); zhujin6319@126.com (J.Z.)

**Keywords:** ripple fixed pattern noise, nonuniformity correction, fuzzy classification, FPGA

## Abstract

Cooled infrared detector arrays always suffer from undesired ripple residual nonuniformity (RNU) in sky scene observations. The ripple residual nonuniformity seriously affects the imaging quality, especially for small target detection. It is difficult to eliminate it using the calibration-based techniques and the current scene-based nonuniformity algorithms. In this paper, we present a modified temporal high-pass nonuniformity correction algorithm using fuzzy scene classification. The fuzzy scene classification is designed to control the correction threshold so that the algorithm can remove ripple RNU without degrading the scene details. We test the algorithm on a real infrared sequence by comparing it to several well-established methods. The result shows that the algorithm has obvious advantages compared with the tested methods in terms of detail conservation and convergence speed for ripple RNU correction. Furthermore, we display our architecture with a prototype built on a Xilinx Virtex-5 XC5VLX50T field-programmable gate array (FPGA), which has two advantages: (1) low resources consumption; and (2) small hardware delay (less than 10 image rows). It has been successfully applied in an actual system.

## 1. Introduction

Imaging systems based on infrared focal plane arrays (IRFPAs) have been widely used in military and civilian applications. However, limited by the quality of the manufacturing of materials and the level of processing technology, the responsivity of the individual photodetectors will vary from detector to detector in the focal plane array. This is termed as the nonuniformity of an IRFPA and is also called fixed pattern noise (FPN). Nonuniformity severely corrupts infrared images and must be corrected in practical applications. In this case, many nonuniformity correction (NUC) techniques have been proposed to compensate for FPN. The NUC techniques are categorized into two classes, namely, calibration-based and scene-based techniques.

**Figure 1:**
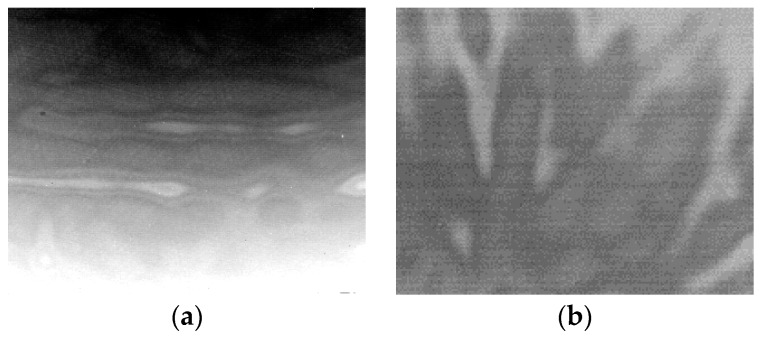
Long-wave infrared image with ripple residual nonuniformity (RNU): (**a**) near-ground sky; and (**b**) deep sky.

Calibration-based nonuniformity correction (CBNUC) algorithms are generally used to reduce FPN, where the simplest and most-used one is two-point calibration [1]. However, the residual nonuniformity will show up when the observed scene radiation exceeds the calibration range [2,3]. Especially for the cooled detector, due to the defects caused by the detector manufacturing, the residual nonuniformity is spatially continuous when observing the low-radiation sky and the shape is ripple-like, as shown in Figure 1. To make matters worse, the residual nonuniformity slowly varies over time. This noise seriously disrupts the detection, identification, and tracking of the sky targets. The calibration-based NUC methods must often be repeated in order to remove the drift noise, necessarily halting the normal operation of the system to allow the use of the blackbody sources, interrupting the scene anyway.

In order to overcome the shortcomings of CBNUC, many scene-based nonuniformity correction (SBNUC) algorithms have been proposed. These techniques only make use of the readout infrared data captured during the normal operation of the imaging system, reducing the optical setup complexity, and avoiding scene interruptions. They are generally identified by two main approaches, namely, statistical methods and registration-based methods. The registration-based algorithms estimate the incident radiation value based on the assumption that the response of the same scene at different pixels should be the same, and achieve a good correction effect in some cases [4,5]. 

However, because of the high computational complexity and large storage demands, the registration-based algorithms are not practical. The statistical algorithms usually make some spatiotemporal assumptions on the irradiance collected by each detector in the array. These approaches are divided into neural network (NN) algorithms [6], temporal high-pass (THP) algorithms [6], and constant-statistical (CS) algorithms [7]. They have a common problem of ghosting artifacts. In order to reduce the ghosting artifacts, Vera et al. improved Scribner’s retina-like neural network approach method using an adaptive learning rate [8]. Hardie et al. proposed a deghosting method based on the temporal gate [9]. This method avoids the accumulation of incident radiation estimation error in the long motion pause situation by gating the update of the NUC parameters. Fan et al. proposed a combined temporal and spatial deghosting technique in the NN-NUC to compensate for the shortcomings of the Hardie’s algorithm [10]. Qian et al. improved the temporal high-pass algorithm by combining it with a spatial low-pass filter [11]. Harris and Chiang developed a strategy to eliminate the ghosting artifact in the CS-NUC method, which is also applicable to the THP-NUC methods [12]. Jin et al. proposed the temporal high-pass nonuniformity correction algorithm based on grayscale mapping (THP & GM) [13]. By using the selective iterative smooth method, the correction speed is greatly improved. However, since the ripple RNU is spatially continuous and only occurs in the sky image, it is difficult to remove it with these algorithms without causing image degradation. 

To achieve the constraints for real-time video, many works integrate NUC algorithms with the sensing technology, using architectures based on field-programmable gate arrays (FPGA), in cooperation with external or embedded processors. Toczek et al. implemented an improved CS algorithm in hardware to perform offset parameter correction of CMOS visible light sensor images [14]. A Xilinx Spartan-6 LX150T was used to process 30 frames per second (fps) video frames of 1280 × 800 pixels on line. Celedón et al. implemented Scribner’s neural network algorithm to the Xilinx Spartan XC3S1200E FPGA to achieve nonuniformity correction of 320 × 240 pixels at 130 fps [15]. The power consumption of the processing platform was 329 mW. Redlich et al. mapped a deghosting constant statistical algorithm onto a Xilinx Spartan-6 XC6SLX45 FPGA platform to implement the NUC for a 640 × 480 pixels, 238 fps infrared imager [16]. Jin et al. presented a Xilinx Virtex-5 XC5VSX50T FPGA implementation of the THP & GM algorithm [13]. The processing delay is less than 10 image rows of pixel cycles.

As far as we know, very limited research has been presented to remove the ripple RNU. Basically, the low-spatial frequency ripple RNU present in sky images requires the NUC algorithm to be aggressive in order to perform correction in a reasonable time. Especially in the field of sky small target detection, the rapid and effective removal of these noises without detail degradation is needed. The current statistically-based SBNUC algorithms, as mentioned before, are often aimed at some high-spatial frequency noise [6,7,8,9,10] and require hundreds to thousands of frames to reach convergence [11]. In our previous research, we verified that the THP & GM algorithm can remove the ripple RNU quickly [13]. However, it will produce detail degradation and ghosting artifacts in non-sky images with distinguishable objects. This is because the threshold values in the algorithm are difficult to select and cannot be adjusted according to the scene. 

In this paper, we propose an improved THP & GM (ITHP & GM) based on scene classification. Since the ripple RNU appears only in the sky image, the scene classifier is used to compute the sky similarity of the image, and then the results adaptively control the correction thresholds to avoid the degradation and ghosting artifacts. The algorithm is implemented on a Xilinx Virtex-5 XC5VLX50T FPGA (Xilinx Inc., San Jose, CA, USA) which can read an IR video stream from the LVDS output of an image storage platform or a long-wave infrared imager and display the processed video on an external monitor using a PAL interface. 

The rest of the paper is organized as follows: Section 2 is dedicated to a detailed description of the proposed algorithm along with highlighting the improvements made and its real-time implementation. In Section 3, the performance of the proposed method is demonstrated by employing infrared video sequences with ripple RNU in PC and FPGA platforms. A brief discussion is presented in Section 4, and the conclusion is summed up in Section 5.

## 2. Methods

### 2.1. The Correction Process

A linear model is usually used to represent the response characteristics of a focal plane array. The measured readout signal $y_{i,j}$ at a given time *n* and the two-point calibration can be expressed as:

(1) $$y_{i,j}\left(n\right)=G_{i,j}\left(n\right)x_{i,j}\left(n\right)+O_{i,j}\left(n\right)$$

(2) $$Y_{i,j}\left(n\right)=\frac{1}{G_{i,j}\left(n\right)}y_{i,j}\left(n\right)-\frac{O_{i,j}\left(n\right)}{G_{i,j}\left(n\right)}$$

where $Y_{i,j}\left(n\right)$ is the result of the calibration at the *ij*th detector, $x_{i,j}$ is the irradiance collected by this detector, $G_{i,j}\left(n\right)$ and $O_{i,j}\left(n\right)$ are the respective gain and bias of the detector. After the blackbody calibration, each unit of the detector has the same response to the same amount of radiation, assuming the linearity of the sensor. However, the correction parameters do not work when the environmental conditions change or the radiation is very low, as well as beyond the calibration range (such as the sky radiation). The THP & GM combines the temporal high-pass filter and spatial low-pass filter and uses a one-point correction model to correct these residual nonuniformity:

(3) $${\hat{y}}_{i,j}\left(n\right)=Y_{i,j}\left(n\right)+{\hat{O}}_{i,j}$$

where ${\hat{O}}_{i,j}$ is the offset correction parameter of the *ij*th detector, and ${\hat{y}}_{i,j}$ is the result of the THP & GM.

**Figure 2:**
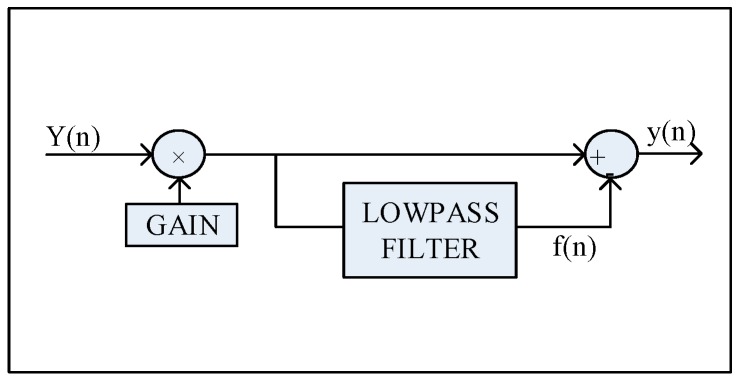
Temporal high-pass filter for adaptive nonuniformity correction (one filter per pixel).

Scribner’s THP-NUC algorithm uses a temporal low-pass filter and differences this result with the input signal to obtain the temporal high-pass filter [6]. The full implementation of the THP-NUC is shown in Figure 2. The filter inherently attenuates low temporal frequencies from the output of each detector on an individual basis and conversely passes high temporal frequencies.

The idea of the THP is to remove the fixed pattern noise. The THP & GM believes the high-frequency component in the time domain is the object, requiring weak correction. On the contrary, the low-frequency component is noise, and it requires iterative correction. The iterative correction model is given by:

(4) $$\begin{array}{l} \hat{O}\left(n\right)=f\left(n\right)-Y\left(n\right)\\ \hat{O}\left(n+1\right)=\hat{O}\left(n\right) \end{array}$$

where *f*(*n*) is the result of spatial low-pass filter. If one pixel in the adjacent frames has a large grayscale difference, it will be regarded as the object which is a short-term high-frequency component in time domain. The THP & GM interrupts such a pixel’s iterative correction to avoid ghosting artifacts. This is the idea of temporal high-pass filter in the THP & GM. Thus, the THP & GM modifies the correction matrix by a temporal threshold as follows:

(5) $${\hat{O}}_{i,j}\left(n+1\right)=\{\begin{array}{cc} 0 & \left|Y_{i,j}\left(n+1\right)-Y_{i,j}\left(n\right)\right|\geq T_{te}\\ {\hat{O}}_{i,j}\left(n\right) & \left|Y_{i,j}(n+1)-Y_{i,j}(n)\right|<T_{te} \end{array}$$

where $T_{te}$ is the temporal threshold. If a large *T_te_* is used, the ${\hat{O}}_{i,j}\left(n+1\right)$ is rarely initialized to 0 and the pixel will be iteratively blurred. On the contrary, the iteration correction is difficult to sustain.

In addition, the process of iterative correction easily causes the burn-in artifacts on the static scene details. The THP & GM uses a low-pass filter with a spatial threshold to protect the details of the image. It removes the pixels which have large grayscale differences with the center pixel in the filter window, this is similar to the bilateral filter, but simpler. The filter can be written as:

(6) $$f_{i,j}\left(n\right)=\frac{\sum_{w=-v}^{v}\sum_{h=-v}^{v}\delta_{i+w,j+h}\left(n\right)\cdot {\hat{y}}_{i+w,j+h}\left(n\right)}{\sum_{w=-v}^{v}\sum_{h=-v}^{v}\delta_{i+w,j+h}\left(n\right)}$$

where *v* is the filter size, *δ* is a selective factor which is defined as follows:

(7) $$\delta_{i+w,j+h}=\{\begin{array}{c} 1\;\left|Y_{i+w,j+h}-Y_{i,j}\right|<T_{sp}\\ 0\;\left|Y_{i+w,j+h}-Y_{i,j}\right|>T_{sp} \end{array}$$

where $T_{sp}$ is the spatial threshold. If a large *T_sp_* is used, the filter mask seems to be larger and will make the filtered image smoother. On the contrary, the detail conservation ability is stronger.

**Figure 3:**
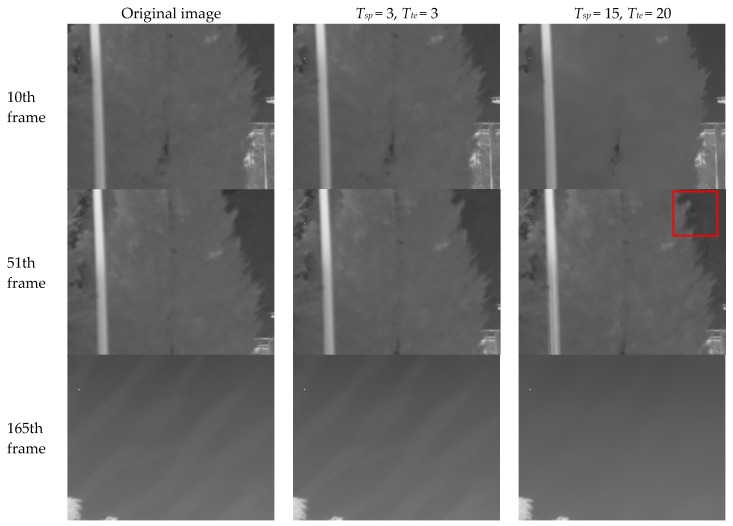
The temporal high-pass nonuniformity correction algorithm based on grayscale mapping (THP & GM) nonuniformity correction (NUC) performance with different threshold values. The red rectangular area shows the ghosting artifacts caused by the large threshold values.

The THP & GM iteratively smooth the temporal low-frequency non-edge pixels, so the fixed pattern noise can be quickly removed. However, the efficiency of the THP & GM depends on the constant value of the threshold. If the noise energy is variable, especially for the spatially-continuous ripple RNU occurring only in the sky image, the adaptation of the thresholds will be challenging. Figure 3 shows the correction results using two different sets of thresholds. The thermal imager remains stationary for first 50 frames and then gradually turned from the ground to the sky.

It can be see that, for the constant large threshold values $T_{sp}=15$ and $\;T_{te}=20$, the details within the spatial threshold, such as the branches in the Figure 3, have been degraded. When the motion resumes, the degraded pixel’s offset correction parameters are not reset due to the large temporal threshold. This causes the ghosting artifacts as shown in the red rectangular area in Figure 3. The constant small threshold values $T_{sp}=3$ and $\;T_{te}=3$ make the spatial filter mask smaller and most pixel’s offset correction parameters equal to 0. This of course protects the details, but does not remove the ripple RNU in the sky. It can be concluded that the larger the threshold values, the stronger the noise removal capability while, at the same time, the weaker the detail conservation ability of the THP & GM algorithm. The thresholds should relate to the spatial and temporal characteristics of the noise.

### 2.2. The Scene Classification

According to the characteristics of the ripple RNU, the ITHP & GM uses the scene classifier to distinguish the sky and the ground scene. Then, the classification results are associated with the thresholds. In other words, as the sky scene in the field of view increases, the threshold values will become larger. In order to protect the details in the image, the threshold values are gradually reduced with the reduction of the sky scene. 

Without loss of generality, we assume that the sky scene appears in the upper rows of the image in the following analysis.

One important feature of the sky image is the gray value is far lower than that for the ground image. It has already been shown that the statistical parameters of sky IR images will vary with the angle of elevation of the imager [17,18,19]. For the near-ground sky image (elevation angle is small), the mean grayscale of each row is typically increasing from the top to the bottom. When the elevation angle increases, the cloudless image gray value has no significant change in the column scan line, but the mean grayscale is far smaller than that for the near-ground image. Another important feature of the sky images are that the gray scale fluctuation is smaller compared to the ground images.

**Figure 4:**
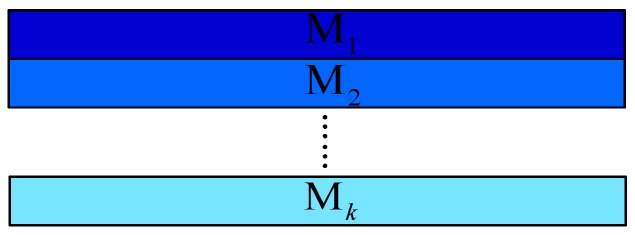
Diagram of the image blocks. Different blocks are represented by different colors.

According to the above characteristics, we assume that the image size is *H* × *W*. The image is divided into *k* blocks, each block has *H*/*k* rows. The mean value of each block is calculated as *M*_1_, *M*_2_ ..., *and M*_k_, as shown in Figure 4. We set the gray level threshold *T*_1_, and count the number of blocks whose gray value is less than *T*_1_ to obtain the parameter A. If A is large, it is considered likely to be a sky image. The grayscale difference between the lower block and the upper block is calculated as *S*_1_ = *M*_2_ − *M*_1_, *S*_2_ = *M*_3_ − *M*_2_ ..., and *S*_k−1_ = *M*_k_ − *M*_k−1_. Parameter B represents the number of these differences greater than 0; if the parameter B is large, then the scene is likely to be the near-ground sky. In order to exclude sky scenes with clouds or other large flying objects, the absolute gray difference between adjacent blocks is used, just like *SS*_1_ = |*M*_2_ − *M*_1_|, *SS*_2_ = |*M*_3_ − *M*_2_| ..., and *SS*_k #x2212;1_ = |*M*_k_ − *M*_k−1_|. The number of these differences that are larger than *T*_2_ is counted as C. If C is large, clouds or significant objects are considered to be present. 

If we deal with the classification by bi-valued Boolean logic to obtain the sky image set, the result can only be true or false, and it is difficult to give a result for the half-sky image. The nonuniformity noise can occur suddenly in this situation due to the inaccuracy of judgment, which limits the application. Inspired by the successful application of fuzzy theory in intelligent control and classification [20,21], we use fuzzy theory [22] to more resiliently establish the gradual transition between the sky scene and the non-sky scene.

**Figure 5:**
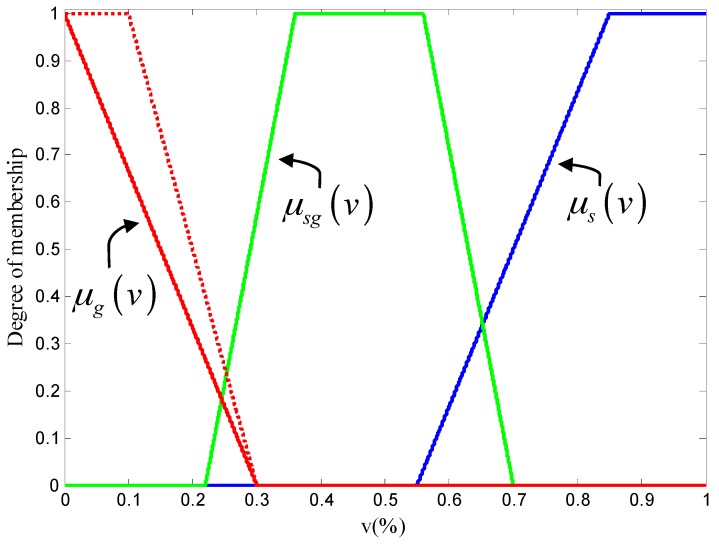
Membership functions of the SKY set (blue line), the HALF-SKY set (green line), and the GROUND set (red line). The red dotted line is the compared membership function of GROUND set.

The sky similarity *v* is defined to denote the extent to which an image resembles a sky image. The greater the *v*, the more the image is similar to the sky image. Let *V* be a set of elements, with a generic element of *V* denoted by *v*. All scenes are divided into three fuzzy sets: the SKY set, the HALF-SKY set, and the GROUND set. The fuzzy sets are characterized by the trapezoidal membership function:

(8) $$\mu \left(v\right)=\{\begin{array}{l} 1-\left(\alpha_{1}-v\right)/\eta_{1}\;\alpha_{1}-\eta_{1}\leq v<\alpha_{1}\\ 1\;\alpha_{1}\leq v<\alpha_{2}\;\\ 1-\left(v-\alpha_{2}\right)/\eta_{2}\;\alpha_{2}\leq v<\alpha_{2}+\eta_{2}\\ 0\;else\; \end{array}$$

where the *α*_1_, *α*_2_, *η*_1_, and *η*_2_ are the four parameters of the function. In our application for membership, we use *α*_1_ = 0, *α*_2_ = 0, *η*_1_ = 0, and *η*_2_ = 0.3 for the GROUND set, denoted as *μ_g_*(*v*); *α*_1_ = 0.36, *α*_2_ = 0.56, *η*_1_ = 0.14, and *η*_2_ = 0.14 for the HALF-SKY set, denoted as *μ_sg_*(*v*); and *α*_1_ = 0.85, *α*_2_ = 1, *η*_1_ = 0.3, and *η*_2_ = 0 for the SKY set, denoted as *μ_s_*(*v*). These membership functions are shown in Figure 5. We can see that in order to make the sky similarity of the ground scene small enough, the *μ_g_*(*v*) curve does not have a portion equal to 1, as shown by the dotted line in the Figure 5.

According to the previous analysis of sky radiation, it can be formalized in the following fuzzy IF-THEN rules:

Rule1: If A is large AND C is small, OR B is large AND C is small, THEN it is a sky image.
Rule2: If A is small AND B is medium AND C is small, THEN it is a half sky image.
Rule3: ELSE it is a ground image.

**Figure 6:**
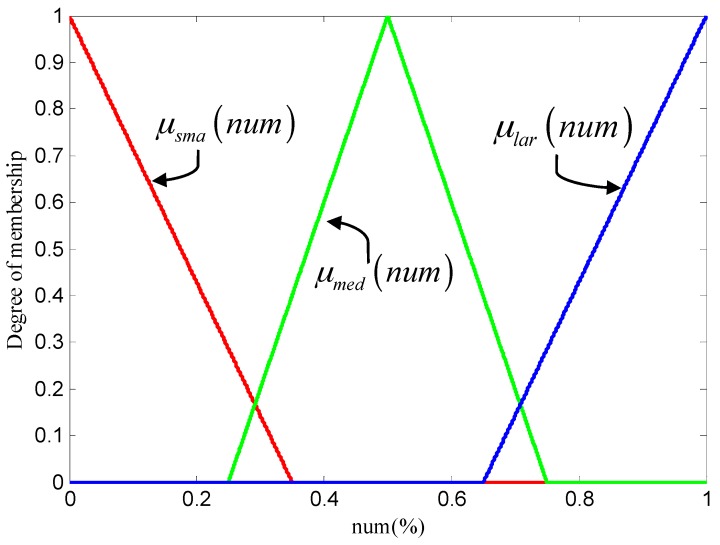
Membership functions of sets SMA (red line), MED (green line), LAR (blue line).

Where the labels small, medium, and large are the fuzzy description of the number. These labels need to be expressed as fuzzy sets. This is achieved by defining membership as functions. We assume that the membership functions of A, B, and C are the same. The function is expressed as:

(9) $$\mu \left(num\right)=\{\begin{array}{c} 1-\left(\alpha -num\right)/\eta \;\alpha -\eta \leq num<\alpha \\ 1-\left(num-\alpha \right)/\eta \;\alpha \leq num\leq \alpha +\eta \\ 0\;else\; \end{array}$$

where *num* is the normalized term, the μ(num) is the triangle membership functions, *α* are the vertices of the triangle function, and *η* is the intersection of the hypotenuse and the *x*-axis. In our application, for the SMA (small) set, the *α =* 0 and *η* = 0.25, for the MED (medium) set, the *α =* 0.5 and *η* = 0.35, and for the LAR (large) set, the *α =* 1 and *η* = 0.35. These membership functions are denoted as *μ_sma_*(*num*), *μ_med_*(*num*), and *μ_lar_*(*num*) and are shown in Figure 6.

Before applying rule inference, the antecedent of each rule needs to be processed using the max or min operation (OR or AND) in order to yield a single value *λ*. The *λ* of each rule is used to provide the output corresponding to that rule. We use AND for implication, which chips the corresponding output membership function at the value *λ*. Since the output of each rule is a fuzzy set, we need to combine the fuzzy sets using the OR operation to yield a single fuzzy output set *Q*. Finally, a single value *v*_out_ representing sky similarity is obtained by calculating the center of gravity of *Q*.

(10) $$v_{out}=\sum_{v=0}^{1}v\cdot Q\left(v\right)/\sum_{v=0}^{1}Q\left(v\right)$$

**Figure 7:**
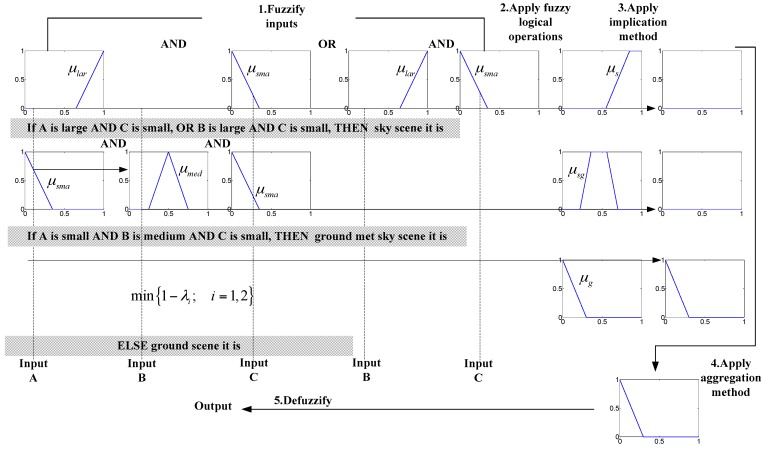
An example of a fuzzy classifier.

The whole process is shown in Figure 7, the gray background section corresponds to the fuzzy rule expression.

### 2.3. Adaptive Threshold

Since the ripple RNU is spatially continuous, it can only be removed by iterative correction using as smooth a filter as possible. The RNU of the ground scene is almost invisible, and it is necessary to stop the nonuniformity correction to avoid “ghosting” and detail degradation. We use the previously-calculated sky similarity to adaptively control the thresholds and the large threshold values are only used in the sky image. The expression is as follows:

(11) $$T_{te}=P_{te}v_{out}$$

(12) $$T_{sp}=P_{sp}v_{out}$$

where $P_{te}$ and $P_{sp}$ are constant parameters. Obviously, the fuzzy classifier has different sky similarities for different scenes. If the image is more like a sky scene, both the values of temporal and spatial thresholds will be increased. Thus, more pixels will be involved in the iterative correction to ensure the rapid removal of sky ripple FPN. Otherwise, the threshold values will become smaller to limit the iterative correction. This is significantly different from the THP & GM.

### 2.4. Hardware Implementation 

**Figure 8:**
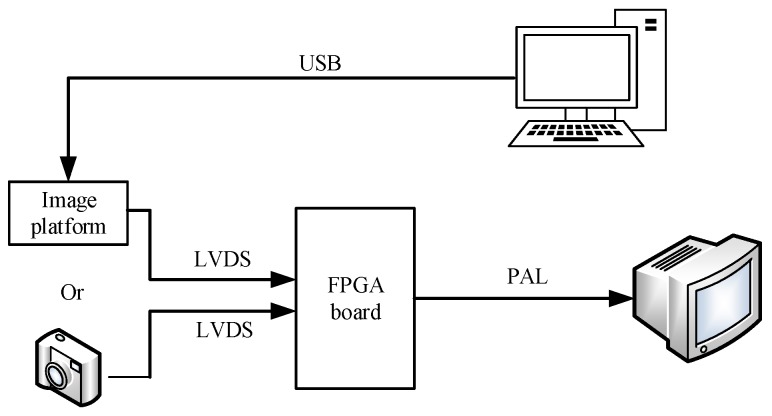
Field-programmable gate array (FPGA) hardware processing system.

The image processing board of infrared imager generally complete the blind pixel replacement, nonuniformity correction and dynamic range compression. Nonuniformity correction, as a pre-processing of the infrared imaging system, needs to be embedded in the detector. As a parallel processing logic device, FPGAs are widely used in infrared detector data preprocessing. In this paper, the hardware platform uses a Xilinx XC5VLX50T-FPGA as the core processing device. Peripheral devices contain 2 × 9 Mb SRAM that can facilitate the realization of the algorithm. The platform includes analog video output (PAL), an RS422 communication interface, a three-way LVDS interface, and a 38-bit general-purpose digital interface (which can be customized according to the request signal). To facilitate the testing of hardware algorithms, we have made an image storage platform that can receive a variety of detector images transmitted by a computer via USB and transfer them to the processing board in LVDS format. The FPGA processing board can not only be connected directly to the detector, but can also be connected to the image storage platform, the whole system diagram is shown in Figure 8.

The algorithm implementation has been conducted in the Verilog hardware description language. It mainly includes adaptive threshold calculation and nonuniformity correction. The calculation of the adaptive threshold is concentrated in the scene classification. The operation of the nonuniformity correction module on one pixel includes reading the same position pixel value of the previous frame, spatial filtering, storing the current frame offset matrix, reading the previous frame offset matrix, modifying the correction matrix, and one point correction, whereas the real-time read and write is done with an external SRAM. The key step is the spatial filtering and the scene classification.

**Figure 9:**
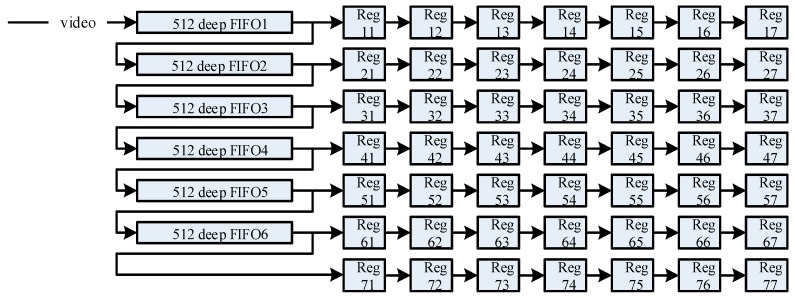
Diagram of filtering window in the FPGA.

The spatial filter mask is generated by the FIFOs in cooperation with the registers. The depth of each FIFO should not be less than the column of the image and the read width is always 16 bits for the infrared imager. As shown in Figure 9, for a 7 × 7 window, six FIFOs are used to connect to each other and cascade with seven sets of Regs (registers). In this way, the data in the FIFOs and Regs are shifted backwards with each pixel clock, which is like a filter mask that automatically swipes to the right pixel by pixel. The 7 × 7 Regs are the filter mask and the data in Reg44 is the center pixel of the window. The filtering operation is performed in a synchronized clock which is eight times faster than the pixel clock; that is, we can manipulate one pixel eight times before it changes, which includes the logic operation and the calculation operations in the filtering. Of course, this part, and the calibration process, are carried out in parallel, and we only need to align the filtering results and the offset value calculation in the timing.

**Figure 10:**
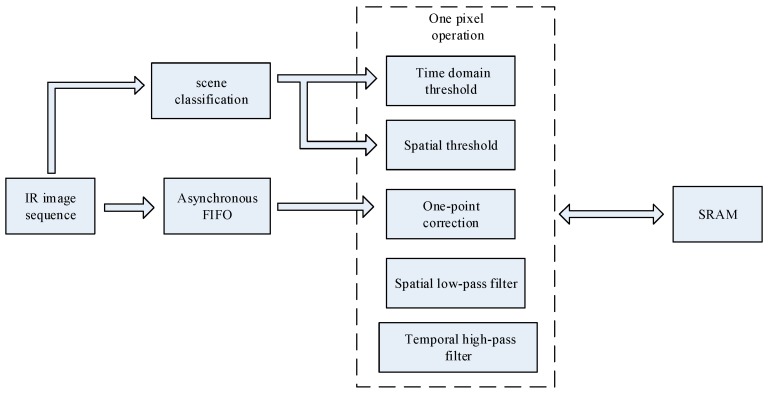
A conceptual schematic of hardware architecture.

The A, B, and C parameters in the scene classification module are calculated in parallel with the calibration process. Then the calculation of the sky similarity by the fuzzy classifier is carried out during the inter-frame period and, due to the linear membership function, it is not difficult to implement with the hardware multiplier. In this process, the image information of the current frame is used to control the correction thresholds for the next frame. The entire algorithm processing architecture is shown in Figure 10.

## 3. Results

In this part, we first carried out scene classification experiments on a series of infrared images to test the accuracy of the fuzzy classifier. Then the proposed algorithm, and some other algorithms, were tested on the real infrared sequence with ripple RNU. Finally, we test the FPGA resource consumption of the algorithm and the real-time processing performance.

### 3.1. Scene Classification Experiment 

**Figure 11:**
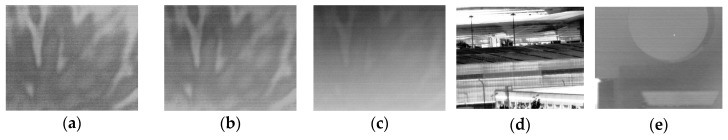

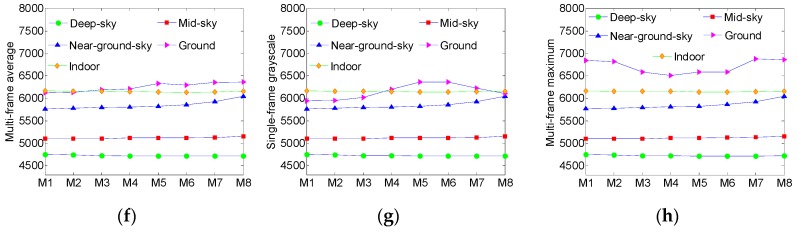
Statistical properties of several scenes. (**a**) Deep-sky image; (**b**) mid-sky image; (**c**) near-ground-sky image; (**d**) ground scene image; (**e**) indoor scene image; (**f**) multi-frame average of blocks; (**g**) single-frame grayscale of blocks; and (**h**) multi-frame maximum grayscale of blocks.

The deep-sky, mid-sky, near-ground-sky, ground, and indoor scenes were imaged in August 2015 using a calibrated infrared imager with a 320 × 256 pixels detector, operating in the long-wave spectral window. The typical images are shown in Figure 11a–e. Each type of scene has 5000 frames. According to our method, the image is divided into eight blocks, each containing 32 rows. Figure 11f–h shows the grayscale characteristics of these images. The X-axis of the curve is the block and the Y-axis is the multi-frame average grayscale (Figure 11f), single-frame grayscale (Figure 11g) and multi-frame maximum gray-scale (Figure 11h) variations along the vertical scanning line. It is clear from the figure that the gray mean value of deep-sky and mid-sky images are at least 1000 lower than that of the ground images, that is, the sky image without the sun in the field of view can be roughly distinguished from the ground image by the gray level threshold. The near-ground-sky curves are ascending from top to bottom on the vertical scanning line in terms of single-frame grayscale, multi-frame average grayscale, and multi-frame maximum grayscale. 

In order to verify the accuracy of the fuzzy classifier, we assume that the images with *v*_out_ between 0.7 and 1 are sky images, between 0.4 and 0.7 are half-sky images, and between 0 and 0.4 are ground images. Another image sequence of 9044 frames was collected in June 2016 using the same imager. These frames were artificially identified as: five sets of all-sky scenes totaling 2995 images, five sets of half-sky scenes totaling 1200 images, and four sets of ground scenes totaling 4849 frames. Each set of images was stored in a DAT file, so each file was the same kind of scene. Then our MATLAB (MathWorks, Natick, MA, USA) program (*T*_1_ = 5300, *T*_2_ = 40) read images in the files and give the classification results, and the accuracy of the classification was calculated, as shown in Table 1.

As can be seen from Table 1, the classification accuracy of the ground images is the highest. It is because the mean grayscale of each block in ground images was significantly higher than that in the sky images or the half-sky images, and also the grayscale variation is larger. This is the basis of the detail conservation of the algorithm for the ground images. The classification accuracy of the half-sky images is lowest and it is easy to mistake the half-sky images as the sky images. This is because the radiation of the distant buildings at the bottom of the image are weak, and the grayscale differences between the buildings and the sky are not large. At this point, B is large and C is small, which just satisfy Rule 1. By reducing *T*_2_ we can avoid this situation as much as possible.

### 3.2. Nonuniformity Correction on Real Infrared Data

We test the effectiveness of the algorithm for ripple RNU using real infrared image sequences. The imager is equipped with a 320 × 256 long-wave focal plane array producing 16 bits of data at 50 fps and had been calibrated by two-point correction. It moved from bottom to top facing the outdoor scenes and the ripple RNU occurs at the 260th frame. There were 450 frames in total. 

**Figure 12:**
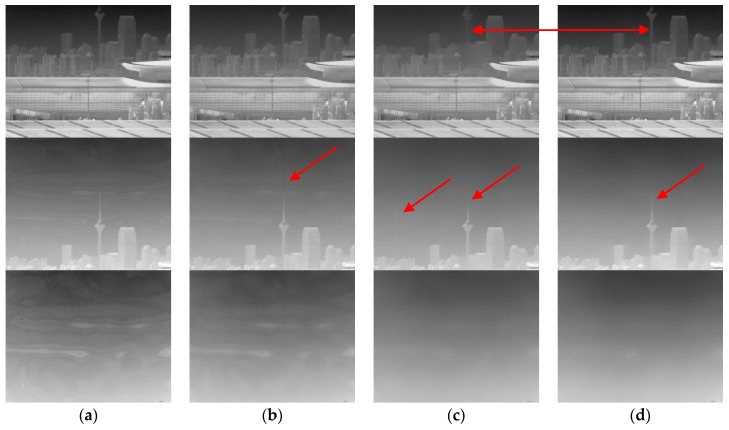
NUC performance comparison of the infrared sequence. (**a**) The original image; (**b**) ST-LMS; (**c**) THP & GM; and (**d**) our method. The upper row corresponds to frame 106, the middle row corresponds to frame 290, and the bottom row is frame 390.

Figure 12a show several typical frames. In the experiment, we used the state-of-the-art Fan’s combined temporal and spatial NN-NUC, denoted as ST-LMS [10], Jin’s THP & GM, and the algorithm proposed in this paper to process the image sequence. All of the algorithm special filters used a 7 × 7 window. The *k*_alr_ in ST-LMS was 10^−6^, the temporal threshold value was 10, and the spatial threshold value was 15. The THP & GM used $T_{sp}=10,\;T_{te}=8$. The parameters of ITHP & GM were set as $P_{te}=15,\;P_{sp}=20,T_{1}=9200,\;T_{2}=40$. The processed images are shown in Figure 12b–d.

**Figure 13:**
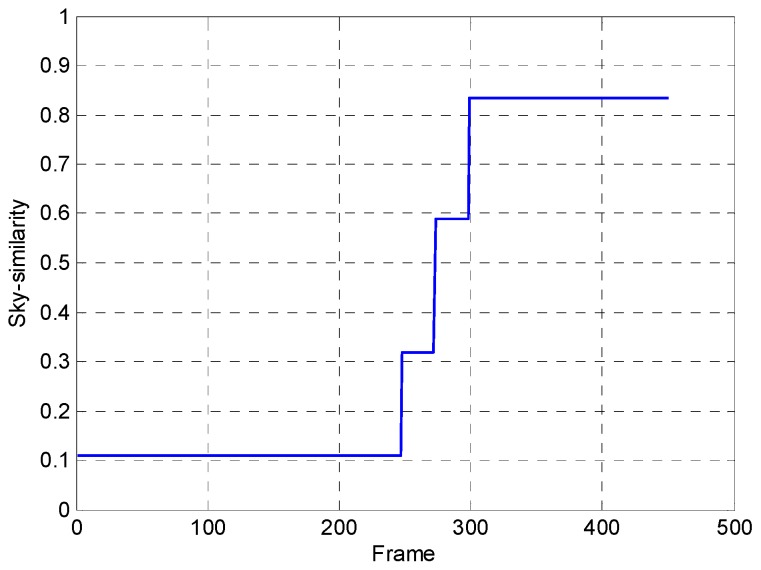
The sky similarity varies with the frames.

From Figure 12b we can see the denoising effect, in which ST-LMS algorithm dealing with the ripple RNU is not obvious within the 390 frames, and the large window size has caused ghosting artifacts, as shown in the area indicated by the red arrow. The THP & GM algorithm has virtually eliminated the ripple RNU at 290 frames. However, the image details are lost at the same time and the ghosting artifacts also appear, as shown in the area indicated by the red arrow in Figure 12c. The ITHP & GM achieves the same noise elimination ability as the THP & GM, as shown in Figure 12d, but the detail in the corrected image is improved as shown in the area indicated by the red arrow in Figure 12d and there are no ghosting artifacts. This is because the THP & GM uses constant large thresholds so that iterative correction is never stopped from the first frame to the end. However, since the adaptive threshold is used in the ITHP & GM, when the pitch angle of the camera increases gradually, the threshold values of the algorithm gradually increase until the sky is full in the view, as shown in Figure 13. It can be seen from Figure 13 that ITHP & GM used thresholds close to 0 in the first 240 frames, which limits the iterative correction. Thus, the image details are almost conserved. The whole process of correction is shown in video S1 (Supplementary Material).

**Figure 14:**
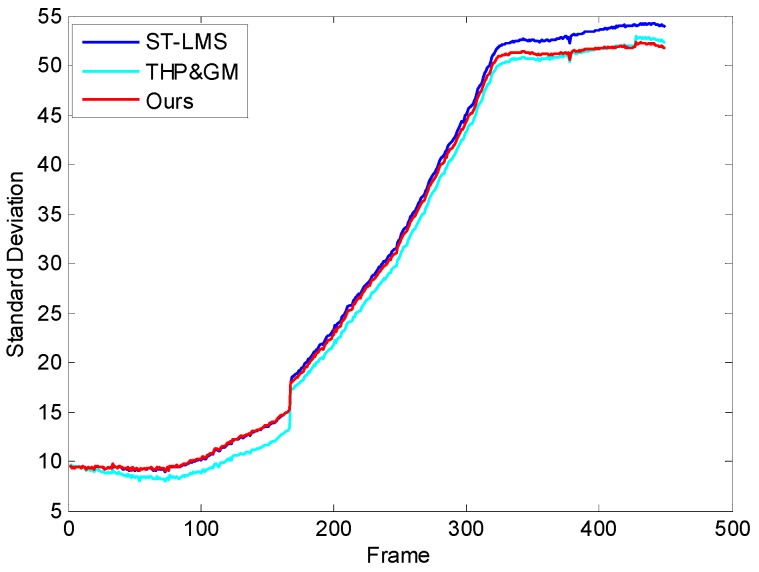
Standard deviation (SD) performance of the tested algorithms.

The visual effect can accurately evaluate the ability of the algorithm to suppress the “ghosting” and “degradation”, but it is not thorough enough to validate the performance of our approach and, thus, the standard deviation (SD) [23,24] of the background pixels of the corrected images are compared. Since the pixel values of the sky background are lower than that of the image scenes, the background pixels can be roughly obtained in these images. The SD curve of the tested algorithms is displayed in Figure 14. It can be seen that the background SD value of the THP & GM is the lowest in the first 168 stationary images, and the ITHP & GM has almost the same SD value as the ST-LMS. This shows that the THP & GM has already started to smooth the image aggressively. Additionally, some of the details within the threshold have been degraded. The ITHP & GM used a small threshold to protect the details at this time. As the camera began to increase the elevation angle, the sky background ripple RNU appears, and the SD curve of the ITHP & GM gradually decreases and reaches an equivalent value with the THP & GM. However, the SD curve of the ST-LMS remains high during this process and the ripple RNU has not been removed. The experimental results further confirm the ripple RNU reduction ability and suppression ability of degradation and ghosting of the ITHP & GM. 

To illustrate the algorithm correction convergence rate on the ripple RNU, the same imager as the first experiment was used to observe the cloudless sky, which has almost uniform radiation and 520 pure sky images were obtained. Some of these images contain small flying objects. The tested algorithms were used to process the sequence. The parameters of the ITHP & GM were *T*_1_ = 5300, *T*_2_ = 40. The other parameters were the same as in second experiment. Then the residual nonuniformity [10,25] after correction is computed as follows:

(13) $$NU=\frac{1}{GV_{avg}}\sqrt{\frac{1}{HW-\left(a+b\right)}\sum_{i=1}^{H}\sum_{j=1}^{W}{\left(GV_{i,j}-GV_{avg}\right)}^{2}}$$

where *a* is the overheating pixel and *b* is the bad pixel. *GV_i_*_,*j*_ is the *ij*th grayscale and *GV_avg_* is the average grayscale. The sum does not include the invalid pixel, where the dead pixel and the overheating pixel are defined based on the same average response rate as the standard blackbody radiation. In the calculation process, it is difficult to define the dead pixel and the overheating pixel because there is no uniform background, so the invalid pixels are omitted.

**Figure 15:**
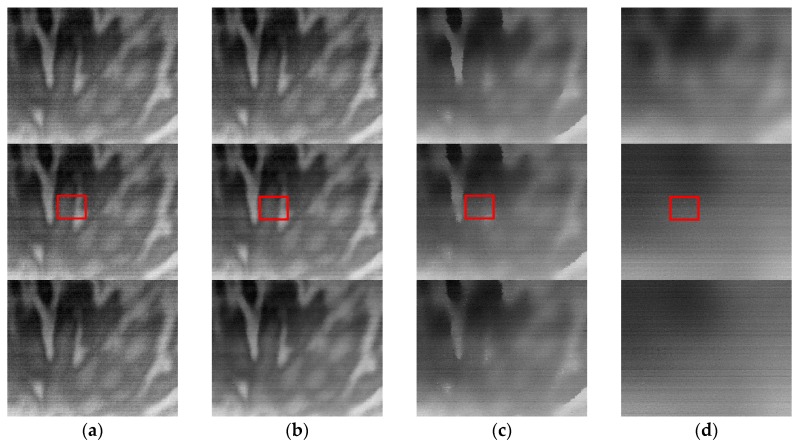
The NUC performance for the pure sky scene: (**a**) original image; (**b**) ST-LMS; (**c**) THP & GM; (**d**) our method. The first row corresponds to frame 50. The second row corresponds to frame 200. The third row corresponds to frame 400. The red rectangular area shows the small targets in the sky.

**Figure 16:**
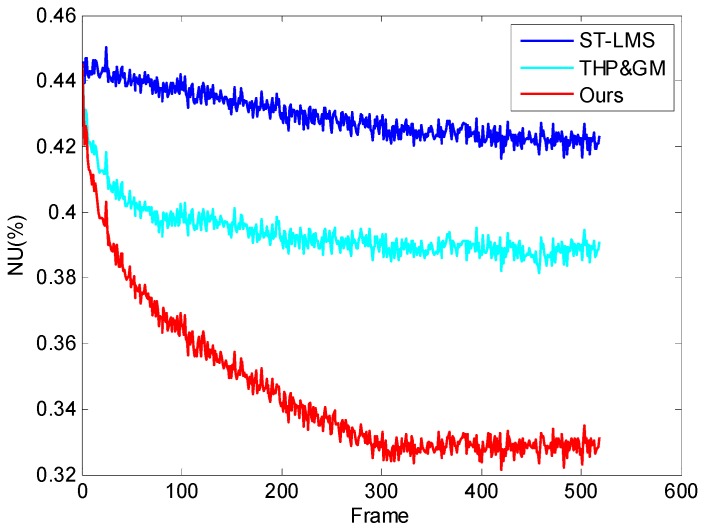
The NU curve of the frames.

Figure 15 shows frames 50, 100, and 400 of the sequences processed by the tested algorithms. The corresponding NU curves are shown in Figure 16. The measurement cannot be accurately analyzed due to the approximation of the cloudless sky as the uniform radiation source. However, the qualitative analysis shows the three residual NU curves have been in a downtrend, and the correction speed of our method is the fastest in terms of the visual effect in Figure 15 and the NU curve in Figure 16. This is mainly because our thresholds ($P_{te}=15,\;P_{sp}=20$) are larger than the THP & GM’s ($\;T_{sp}=10,\;T_{te}=8$) under the control of the scene classifier. On the other hand, the ST-LMS has little effect on the ripple RNU. In addition, from the red rectangular area in the second row of Figure 15, it can be seen that the image processed by our method displays the small target more obviously, which can be more clearly seen in video S2 (Supplementary Material). This experiment shows that the thresholds of our algorithm are more confident than that of the THP & GM, which makes the removal of ripple RNU more efficient.

### 3.3. Real-Time Performance

The pixel clock of the 320 × 256 array detector used here was 15 MHz and the frame rate was 50 fps. The 15 MHz asynchronous clock, and the 120 MHz pixel operation clock were generated with a phase lock loop (PLL) through the onboard 50 MHz clock. The asynchronous FIFO was used to buffer the input data and obtained the image data synchronized with the onboard operation clock. The resource consumption of the whole algorithm is shown in Table 2, the FPGA internal resources mainly used in the module are: six 16-bit wide and 512 deep FIFOs, seven DSP48E (25 × 18-bits) multiplier, and 1600 slices. The phase difference between the rising edge of the field sync signal of the processed image and the unprocessed image was measured with an oscilloscope. The result is 77 μs, which is only 0.3% of the one frame period. The resource cost of the algorithm is not large, and it can be ported to other infrared detector processing boards. 

The worst case analysis of timing parameters was performed for all modules implemented in the FPGA device. A timing analyzer summary revealed that the maximal total delay introduced by combinational circuits is 6.035 ns, and the theoretical maximum frequency of the module is 165.7 MHz. Since we used an operational clock which is eight times faster than the pixel frequency, the highest pixel clock frequency supported by our design is 20 MHz. Replacing the multiplier frequency operation with a pipelined architecture can increase the frequency that can be supported, but this also increases the latency of processing. In addition, for inter-frame operations, only 200 pixel clock cycles are occupied. These two parameters provide a reference for the frame rate range of detectors that can be supported by this design. 

**Figure 17:**
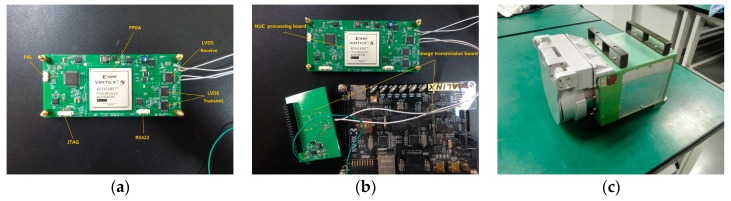
Photographs of the physical system. (**a**) FPGA processing board; (**b**) FPGA processing board and image storage board; and (**c**) the thermal imager equipped with the processing board.

The system physical diagram is shown in Figure 17. A camera-link transfer board was used to connect with the FPGA processing board by the 38-bit GPIO to capture the real-time processed video as shown in Video S3 (Supplementary Material). Among them, 100 images in the image memory board are sent repeatedly as the data source. The effect is the same as that processed by the PC.

## 4. Discussion

As demonstrated in the previous section through the different experiments, our method outperformed the tested methods in terms of the speed of convergence and the detail degradation and ghosting artifacts suppression. This benefits from the adaptive thresholds in the algorithm.

**Figure 18:**
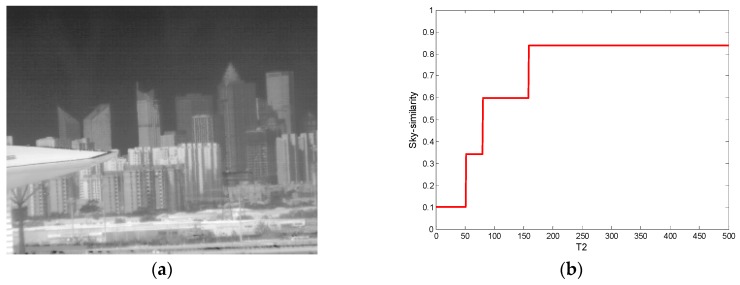
Sky similarity with different *T*_2_ for half-sky image. (**a**) A half-sky image; and (**b**) the corresponding sky similarity curve.

Since the temporal threshold and the spatial threshold of the algorithm are controlled by the fuzzy classifier, the NUC performance is mainly determined by the classification result. The first experiment shows that the recognition accuracy of the half-sky image is the lowest. This will affect the performance of the correction. If the calculated sky similarity for these images is too large, it will be judged as a sky image. Then the use of large thresholds in the NUC process will cause some of the details at the bottom of the image to be lost. On the contrary, if the calculated sky similarity is too small, a small threshold will protect details, but at this time the ripple RNU cannot be removed. One can adjust the sky similarity of such scenes by changing the value of *T*_2_. If the detector does not suffer from ripple RNU in half-sky observations, *T*_2_ should be reduced and so a larger C obtained, and then the calculated sky similarity will be smaller. On the contrary, when the noise is very serious, *T*_2_ should be larger so the calculated sky similarity can yield aggressive thresholds to remove the ripple FPN. Figure 18 shows the sky similarity for a half-sky image with different *T*_2_ values (*T*_1_ = 5300). It is worth noting that since *T*_2_ can only determine the parameter C in the fuzzy operation, the sky similarity does not increase infinitely as *T*_2_ increases.

From experiments 2 and 3, the subjective and objective evaluation show that our algorithm has better ripple RNU removal ability than the tested algorithms. Since the noise is spatial low-frequency and only appears in the sky image, it is difficult for the neural network-based algorithm to estimate the approximation of the incident radiation by a low-pass filter. Thus, the algorithm behaves poorly in the experiment. The THP & GM utilizes the idea of temporal high-pass filter to quickly remove the fixed pattern noise. However, its robustness depends on the threshold values set in the algorithm. It will remove all of the image details within the constant thresholds. The ITHP & GM uses the scene classifier based on fuzzy theory to control the thresholds adaptively. If the image is more like a sky scene, the values of the thresholds are increased. Otherwise, the threshold values become smaller to limit the iterative correction more and, so, the detail conservation ability is improved. By this method our algorithm can remove the ripple RNU quickly while, at the same time, improving the level of detail in the corrected images. The experimental results also show that the images processed by our algorithm are more conducive to the small target detection.

We also implemented the algorithm on a FPGA platform. An important index for investigating the real-time processing ability of algorithms is latency. In our imager, the algorithm processing delay is much smaller than the image frame period. Since the threshold *T*_1_ and *T*_2_ of the scene classifier needs to be set, we specify the operating mode of the camera, that is, the thermal imager needs to be aimed at the sky at the time of booting, and the manual key triggers the processing board to calculate *T*_1_, *T*_2_ through 10 sky images.

## 5. Conclusions

In this paper, we propose an improved THP & GM method to effectively remove ripple RNU. The fuzzy classifier is used to control the thresholds in the algorithm so that they are adjusted adaptively according to the scene. The NUC performance on the real infrared image shows that the proposed algorithm can remove the noise more quickly while preserving more details and suppressing more ghosting artifacts compared with some well-established methods. Then it provides higher-quality infrared image data for the follow-up small target detection. In addition, the algorithm has been ported to the FPGA platform for real-time correction. Furthermore, it has been successfully applied to some domestic infrared camera models.

The algorithm sometimes degrades distant buildings when dealing with half-sky images. This is due to the failure of the classifier. Future work can focus on how to more accurately differentiate the ripple RNU from the scene.

## Figures and Tables

**Table 1:** Recognition accuracy test.

Scene	Sky	Ground	Half Sky
Classification accuracy	98.9%	99.9%	98.3%

**Table 2:** Algorithmic resource consumption.

Type	Slices	Slice Reg	LUTs	LUTRAM	BRAM/FIFO	DSP48E
Used	1600	2000	4000	70	6	7

## Supplementary Materials

The following are available online at http://www.mdpi.com/1424-8220/17/5/1070/s1, Video S1: Ripple RNU correction performance comparison. Video S2: Ripple RNU correction convergence rate test. Video S3: Ripple RNU correction performance test on-line.

## Abbreviations

The following abbreviations are used in this manuscript:

RNU	Residual nonuniformity
FPGA	Field-programmable gate array
IRFPAs	Infrared focal plane arrays
FPN	Fixed pattern noise
NUC	Nonuniformity correction
CBNUC	Calibration-based nonuniformity correction
SBNUC	Scene-based nonuniformity correction
NN	Neural network
THP	Temporal high-pass
CS	Constant-statistical
THP & GM	Temporal high-pass nonuniformity correction algorithm based on grayscale mapping
ITHP & GM	Improved THP & GM
LVDS	Low-Voltage Differential Signaling
PAL	Phase Alteration Line
FIFO	First In First Out
SRAM	Static Random Access Memory
ST-LMS	Least mean square based nonuniformity correction algorithm combined temporal and spatial information
SD	Standard deviation
NU	Nonuniformity
